# Methylated DNA Binding Domain Protein 2 (MBD2) Coordinately Silences Gene Expression through Activation of the MicroRNA *hsa-mir-496* Promoter in Breast Cancer Cell Line

**DOI:** 10.1371/journal.pone.0074009

**Published:** 2013-10-29

**Authors:** Sebastian Alvarado, Joanne Wyglinski, Matthew Suderman, Stephen A. Andrews, Moshe Szyf

**Affiliations:** 1 Department of Pharmacology and Therapeutics, McGill University, Montreal, Quebec, Canada; 2 Sackler Program for Epigenetics and Developmental Psychobiology, McGill University, Montreal, Quebec, Canada; 3 McGill Centre for Bioinformatics, McGill University, Montreal, Quebec, Canada; Institut national de la santé et de la recherche médicale, France

## Abstract

Methylated DNA binding protein 2 (MBD2) binds methylated promoters and suppresses transcription in *cis* through recruitment of a chromatin modification repressor complex. We show here a new mechanism of action for MBD2: suppression of gene expression indirectly through activation of microRNA *hsa-mir-496*. Overexpression of MBD2 in breast epithelial cell line MCF-10A results in induced expression and demethylation of *hsa-mir-496* while depletion of MBD2 in a human breast cancer cell lines MCF-7 and MDA-MB231 results in suppression of *hsa-mir-496*. Activation of *hsa-mir-496* by MBD2 is associated with silencing of several of its target genes while depletion of MBD2 leads to induction of *hsa-mir-496* target genes. Depletion of *hsa-mir-496* by locked nucleic acid (LNA) antisense oligonucleotide leads to activation of these target genes in MBD2 overexpressing cells supporting that *hsa-mir-496* is mediating in part the effects of MBD2 on gene expression. We demonstrate that MBD2 binds the promoter of *hsa-mir-496* in MCF-10A, MCF-7 and MDA-MB-231 cells and that it activates an *in vitro* methylated *hsa-mir-496* promoter driving a CG-less luciferase reporter in a transient transfection assay. The activation of *hsa-mir-496* is associated with reduced methylation of the promoter. Taken together these results describe a novel cascade for gene regulation by DNA methylation whereby activation of a methylated microRNA by MBD2 that is associated with loss of methylation triggers repression of downstream targets.

## Introduction

Covalent modification of cytosine in CpG dinucleotides in 5′regulatory regions of genes by methylation has been shown to regulate gene function in *cis* by suppressing transcription of the juxtaposed gene [1]. However, genome wide analyses of RNA transcription have repeatedly shown only partial correlation between methylation states of promoters and transcription. Most commonly seen are unmethylated promoters that are nevertheless inactive [2]. Remarkably, even pharmacological DNA demethylation does not result uniformly in gene induction but a fraction of the transcriptome is suppressed [3], [4]. This suggests that DNA methylation states are not limited to *cis*-suppression but might be controlling downstream cascades of gene regulatory events.

An accepted hypothesis is that the DNA methylation signal is read by methylated DNA binding proteins that recruit repressive complexes and suppress gene expression *in cis* [5], [6] . Methylated DNA binding protein 2 (MBD2) binds methylated DNA and has been shown to partner with the NuRD complex a multisubunit containing histone deacetylase activities (HDAC) [7], thus promoting gene silencing through inactivation of chromatin configuration [8]. There is a wide body of research that has established the role of MBD2 in methylation dependent *cis*-gene suppression. However, it was also proposed that MBD2 could act as an activator of gene expression either through recruitment of histone acetyl transferases (HAT) and other transcriptional activators such as TACC3 [9] the HTLV-1 TAX1 activator [10] or through promoting DNA demethylation [11], [12] although the DNA demethylation biochemical activity of MBD2 has been contested by several studies [7], [8].

We have previously shown that MBD2 is required for activation and maintaining a demethylated state of prometastatic genes in breast cancer [13] prostate cancer [14] and liver cancer [15] and that inhibition of MBD2 in colorectal and lung cancer cell lines reverses tumor growth as explants *in vivo* [16] [17] and overexpression of MBD2 leads to activation of *Methylated TypeII Hexokinase Gene* in hepatocytes [18]. A conserved sequence required for demethylation of cytokines in mature Th2 cells CNS-1 is bound to MBD2 when these cells undergo demethylation [19]. *mbd2*−/− mice exhibit hypermethylation of certain tumor suppressor genes which is partial in *mbd2*−/+ mice [20]. In this paper, we examined the hypothesis that MBD2 could cause coordinate gene repression through activation of repressive pathways of gene regulation.

Highly-networked candidate repressors in the cell are microRNAs. microRNAs are small 17–22 nt non coding RNAs expressed in several eukaryotic organisms that regulate the stability and processing of target mRNA through direct binding to 3′UTRs [21], [22]. The average microRNA has the potential to bind up to 100 different targets in the cell positioning them as nodal global regulators of disease where transcriptional programs involving multiple genes change drastically [23]. MicroRNAs have been shown to play a role in cell proliferation, differentiation, apoptosis and development causing small reductions in the level of hundreds of target mRNAs for each microRNA [21], [22]. We tested, therefore, the possibility that MBD2 could affect gene repression of network of genes through activation of a methylated microRNA. Although microRNAs have been demonstrated by several studies to be regulated by DNA methylation [24]–[27] chromatin modification [28] and transcriptional regulators [29], the possibility that methylated DNA binding proteins could control expression of a group of genes through changing the methylation state of a microRNA has not been considered. We show here that MBD2 could cause gene repression of a group of genes through activating a microRNA that silences these genes.

## Materials and Methods

### Plasmid Promoter Constructs and *in vitro* methylation of *mir-496*

A PCR amplified fragment (Using primers in Table 1) containing the mir-496 promoter 5′ regulatory region (−315→+161 relative to the TSS) was cloned into PCR2.1 and sub-cloned using HindIII and BamHI restriction sites into the CpG-free pCpGl luciferase reporter [30] in sense and antisense directions. Methylation of promoter constructs was carried out with 2 rounds of methylation with the CG specific *SssI* Methyltransferase and the methyl donor S-adenosylmethionine as recommended by the manufacturer's guidelines (NEB, Cat#. M0226L).

**Table 1:** Primers used for bisulfite mapping and ChIP of mir-496.

Name	Sequence
Outer bisulfite mir-496 forward −184..−162	TGGGTGGTGTGTTGttAttTTt
Outer bisulfite mir-496 reverse −402..−423	TCCATTCAaCCAaaAaTTCCTT
Nested bisulfite mir-496 forward −83..−61	TGGAGGTTGTttATGGTGTGTT
Nested bisulfite mir-496 reverse +368..+389	CCACACAACCAAAATAATTTCA
Outer pcpgl bisulfite mir-496-pcpgl forward	TTAAAAGGAATTttTGtAGGAtTAG
Outer pcpgl bisulfite mir-496-pcpgl reverse	TTTCTTAATATTCTTaaCATCCTCCA
Nested bisulfite mir-496-pcpgl forward	TTTTTTGAATGGTTTTTTGTAAGAG
Nested bisulfite mir-496-pcpgl reverse	AAAAAAAATTAACCATATAATACTCATCAT
Pyrosequencing mir-496-pcpgl S1	AGTAAGGGATGGAGT
Pyrosequencing mir-496-pcpgl S2	TGTTGTTATTTTTTTGATTTTTAGT
ChIP mir-496 −11..+264 for	GGAAGCGAGCACCCAAGT
ChIP mir-496 −11..+264 rev	CATGTCAACTAAAACGTCAGCA
ChIP mir-496 −834..−550 for	GGGTCTGCGCTAGCGTGT
ChIP mir-496 −834..−550 rev	AAGCTCCACTTCTTCCCCAAA
mir-496-pcpgl ChIP forward	GGAAGCGAGCACCCAAGT
mir-496-pcpgl ChIP reverse	TGCCATCTTCCAGAGGGTAG
luc-pcpgl ChIP forward	TCCCTGAAGTTGGTGGAGAC
luc-pcpgl ChIP reverse	GCAGGTGTGGTCAGAGATGA
qPCR MBD2 forward	CAAAGTCACAAATCTCCTAGTAAAGT
qPCR MBD2 reverse	TATAATTTGTTCTGTTACATCTGATACACT
qPCR CTSH forward	TACTGGCTGTTGGGTATGGAG
qPCR CTSH reverse	CGATGAGGAAGTACCCGTTC
qPCR POU2F3 forward	ACTCCAAAGCAGCAGTGAAC
qPCR POU2F3 reverse	CGGTACCAAGATCCTGAAGAG
qPCR PTGS1 forward	CGTAGGAGAGAAGGAGATGG
qPCR PTGS1 reverse	AGAAGCAGTCCAGGGTAGAAC

Expression of targets and MBD2.

### Luciferase Assay

For luciferase assays; HEK293 cells were plated at a density of 1×10^5^ cells per well in 6 well plates. MBD2b and mtMBD2b (with a deletion of the methylated DNA binding domain) were subcloned into the pEF6 (Invitrogen) expression vector from the previously described pcDNA3.1-His-MBD2 [11]. To generate the mutant MBD deleted plasmid, pcDNA3.1-His-MBD2 was digested (KpnI) to remove the MBD domain (nucleotide 601–812) and blunted by Klenow (Roche). Subsequently, the pcDNA3.1 plasmid was digested (NotI) and the released fragment was ligated into pEF6 (Invitrogen). Co-transfections for luciferase assays were performed with CaCl_2_ precipitation as described previously [31] with a total of 2 ug of plasmid transfected using a fixed 200 ng of luciferase promoter and a ratio of 1∶3 of expression construct (MBD2/mtMBD2 to empty backbone, pef6). Constructs were transfected into HEK293 cells, harvested at 72 hours and assayed with the Luciferase Assay System as recommended by the manufacturer's guidelines (Promega, Cat#. E1483). Luciferase activity per condition was normalized to total protein concentration.

### Cell culture and transfections

Human non-invasive breast cancer cells MCF-7, invasive breast cancer cells MDA-MB-231 and nontransformed immortalized breast epithelial cells MCF-10A were purchased from American Type Culture Collection (ATCC, Manassas, Virginia). MCF-7 cells were cultured in minimum Eagle's medium with 10 µg/ml of insulin (Invitrogen, Carlsbad, California). MDA-MB-231 cells were cultured in Dulbecco's modified Eagle's medium (Invitrogen). All media were supplemented with 10% fetal bovine serum, 2 mM glutamine, 100 U/ml penicillin and 100 µg/ml streptomycin. MCF-10A cells were cultured in Mammary Epithelial Basal Media (MEGM®; Lonza/Clonetics Corporation, CC-3150) supplemented with the BulletKit® provided by the manufacturer. The GA-1000 (gentamycin–amphotericin B mix) from the BulletKit® was not added to the media as recommended by ATCC. Transient transfections were carried out using lipofectin (Invitrogen, Cat. #18292-037) as described previously [32] with minor modifications, siRNA was transfected at a final concentration of 70 nM. SiRNAs were selected from one of the siGENOME SMARTpool® sequences provided by Dharmacon, (Lafayette, Colorado). This concentration was optimized in preliminary experiments using transient knockdowns at several concentrations from 20 to 200 nM. Scrambled control was used as control (target sequence ordered: scrambled control 5′-GCCUUGGCAGCCUAGGCGA-3′, and siMBD2 5′-UUACUAGGCAUCAUCUUUCUU-3′). Both Anti-mir-496 (Exiqon, Cat.# 410275-00 5′-AGATTGGCCATGTAATACTC-3′) locked nucleic acids (LNA) and negative control LNA (Exiqon, Cat. # 199004-00 5′GTGTAACACGTCTATACGCCCA-3′) were transfected with lipofectin to a final concentration of 75 nM as described above. Lentiviral infection of MCF-10A was performed as described previously [33]. Briefly, 3×10^6^ HEK293 cells were cultured 24 hours before transfected with 5 ug of pMD2G-VSVG, 5 ug of pCMV-R8.91 and 5 ug of MBD2-lenti plasmid (Open Biosystems) using Fugene (Roche). Cells were washed with PBS and incubated 48 hours with DMEM (Invitrogen). Supernatant was collected and filtered with 0.45 um disk filter and used to infect MCF-10A (plated at 500,000 cells 24 hours prior to infection).

### Expression arrays and validation

RNA was extracted using Trizol according to manufacturer's instructions (Invitrogen, 15596-026). For transcriptome analysis, 1 µg of RNA from MCF-10A cells infected with an MBD2 lentivirus or control empty virus and were subjected to microarray expression analysis using Affymetrix Human Genome U133_Plus 2.0 (Array hybridization was performed at the Génome Québec Innovation Centre, Montréal, Canada). Biological replicates were normalized using the RMA method. Differentially expressed genes were chosen to be those with greater than 1.5-fold increase or less than 0.5-fold decrease in each sample as compared to untreated control. For expression array validation of the microarray results a total of 3 ug of RNA was used for RT-PCR cDNA conversion with AMV reverse transcriptase (Roche, Life Technologies) and random hexamers. Primers were designed to extend across exon boundaries to rule out genomic DNA contamination (Autoprime) and expression was normalized relative to GAPDH expression (primers in Table 1). Quantitative PCR (qPCR) was carried out using the SYBR Green method (Roche, Life Technologies) on LightCycler 480. MicroRNA cDNA conversion was carried out using the Miscript PCR System (Qiagen,Cat #218160). Expression of the mature *mir-496* was measured using miScript primer assays (Qiagen, MS00007707) that spanned the entire mature miRNA and calculated its levels relative to *RNU1A1* noncoding housekeeping RNA control . This control was suggested by the manufacturer to be stable across different conditions. qPCR was carried out using miScript SYBR Green PCR kit (Qiagen, Cat. # 218073). All data was Analyzed using the Absolute Relative Quantification LightCycler 480 software.

### MBD2 Chromatin Immunoprecipitation

MCF-10A infected with MBD2 lentivirus and control were enriched for MBD2-bound DNA through immunoprecipitation as described previously. Briefly, 3 million cells from each cell line was fixed with 1% formaldehyde for 15 minutes at 37°C in the presence of protease inhibitor (Complete, Roche). Fixed cells were then lysed and subjected to sonication. Each sample was pre-cleared with protein G agarose and divided into three sub-samples: Input (100 µl), Bound (900 µl)-to be incubated with 50 ug anti-MBD2 sheep polyclonal antibody at 1 mg/ml (Upstate-Millipore, 07-198), and control-to be incubated with sheep IgG non-specific antibody (negative control, Santa-Cruz Biotechnology, sc-2717) overnight at 4°C. The next day, the unbound fraction was removed, and DNA bound to the beads was subjected to multiple salt washes. Washes were carried out with low salt wash (0.1% SDS, 1% Triton-X, 2 mM EDTA, 20 mM Tris, 150 mM NaCl), high salt(Same as low salt only 500 mM NaCl) , LiCl wash (0.25 M LiCl, 1% NP-40, 1% deoxycholate, 1 mM EDTA, 10 mM Tris pH 8) followed by six TE washes. The bound fractions were then eluted and the antibodies were degraded by protease K treatment. ChIP DNA was used as a template for QPCR.

### Methylated DNA Immunoprecipitation (MeDIP) and Array Hybridization

MCF-10A infected with MBD2 lentivirus and control were enriched for methylated DNA through immunoprecipitation as described previously by Cedar's group [34] For array hybridization, labeled input and bound DNA samples were hybridized to custom designed 244K promoter tiling array (Agilent Technologies) that contained probes covering all transcription start sites at intervals from 800 bp upstream to 200 bp downstream of all genes described in Ensembl (version 44) and within 250 bp of approximately 400 microRNAs from miRBase, all at 100 bp-spacing. The array covered 36,957 transcription start sites corresponding to 18,468 genes. All the steps of hybridization, washing, and scanning were done following the Agilent protocol for ChIP-on-chip analysis. Immunoprecipitation validation was calculated by measuring qPCR of bound over input using primers in Table 1 on Roche LightCycler 480. All microarray data are MIAME compliant and the raw data have been deposited in Gene Expression Omnibus (GEO) at NCBI (www.ncbi.nlm.nih.gov/geo/), accession numbers: GSE47857 (Methylation profiles of MCF-10A cells infected with MBD2 lentivirus) and GSE47873 (Gene expression profiles MCF-10A cells overexpressing MBD2)

### Bisulfite mapping

Bisulfite mapping of DNA was carried out as described previously [35]on a total of 3 ug of DNA. In MCF-10A, MCF-7 and MDA-231, The genomic mir-496 locus was amplified with a nested PCR using primers in Table 1 (n = 50 clones). PCR products were then cloned into PCR2.1 using a TOPO TA cloning kit (Invitrogen, Cat. # K4500-02) and sequenced with a Beckman Coulter CEQ sequencer. Bisulfite mapping of the transiently transfected *hsa-mir-496-pCpGl* Luciferase expression plasmid was carried out using pyrosequencing with a first round of outer PCR primers located within the pCpGl construct (to differentiate between the exogenous transfected promoter and the endogenous promoter) and a second round of inner PCR within the cloned *mir-496* sequence with biotinylated primers in Table 1. Pyrosequencing was carried out directly on the PCR product using a Biotage Q24 Pyrosequencer according to the manufacturer's guidelines. Data was analyzed with Pyromark Q24 Software.

### Statistical Analysis

Statistical analysis for bisulfite mapping and qPCR data was performed using unpaired *t*-tests. Each value represents the mean ± S.E.M. of three independent experiments. The results were considered statistically significant when *P<0.05, **P<.001, ***P<.0001.

## Results

### Over expression of MBD2 triggers demethylation and activation of *hsa-mir-496*

**Figure 1:**
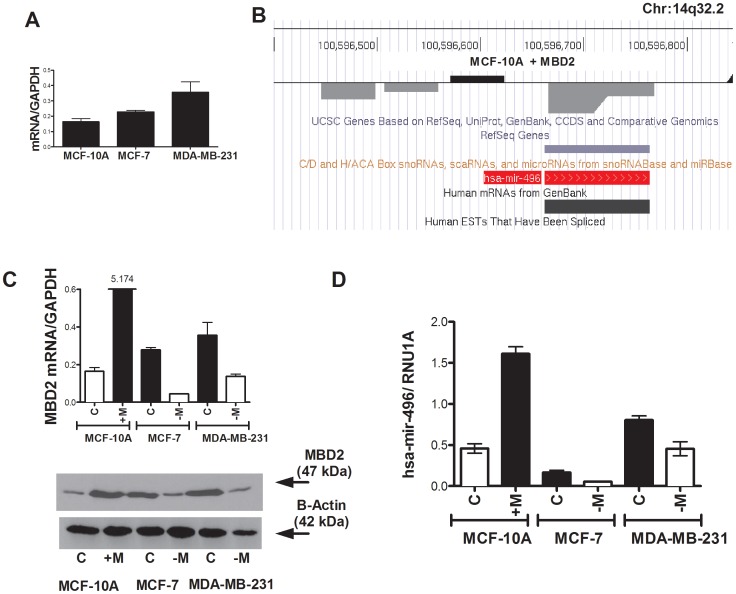
Depletion of MBD2 in mammary breast cancer cell lines leads to induction of *hsa-mir-496* expression. (A) qPCR of endogenous MBD2 mRNA in MCF-10A, MCF-7 and MDA-231. (B) A track showing the position of demethylated probes (descending grey bars) in the *hsa-mir-496* promoter region as determined by MeDIP enrichment for methylated DNA and hybridization to a genome wide promoter array. (C) qPCR of MBD2 mRNA levels in MBD2 transfected (black) MCF-10A and controls (empty), and siRNA-MBD2 treated MCF-7 and MDA-231 cells (empty boxes) and controls (black boxes). Bottom panel is a Western blot analysis with an anti MBD2 antibody (D) qPCR quantification of *hsa-mir-496* in MBD2 transfected (black) MCF-10A and controls (empty), and siRNA-MBD2 treated MCF-7 and MDA-231 cells (empty) and controls (black).

MCF-10A, MCF-7 and MDA-MB-231 mammary cell lines were used in our study since they express varying levels of endogenous MBD2 (Fig. 1A Fig. 1C) ranging from low (MCF-10A) to high, (MDA-MB-231). Deregulation of *MBD2* expression was previously observed in tumors. For example Stefanska et al reported that MBD2 expression is highly elevated in hepatocellular carcinoma biopsies relative to control normal liver tissue. Similarly Billard et al. reported that in invasive breast ductal carcinoma the level of MBD2 expression is significantly associated with tumor size [36]. We then chose the cell line with the lowest level of MBD2 (MCF-10A) (Fig. 1A) as a model system to determine the impact of increased MBD2 expression level on the methylome by overexpressing ectopic MBD2 in these cells (Fig. 1C). This cell line was then subject to methylated DNA immunoprecipitation and array hybridization (and mRNA expression array analysis). From our meDIP array we identified a panel of demethylated microRNAs in response to MBD2 overexpression (Table 2). We focused our study in this paper on *hsa-mir-496* which showed robust demethylation by ectopic expression of MBD2 (Fig. 1 B,D). It is clear however that levels of MBD2 *per se* are not exclusively determining the steady state levels of *hsa-mir-496* since MCF-7 cells express lower levels of *hsa*-*mir-496* (Fig. 1D) than MCF-10A cells despite their higher levels of endogenous MBD2 (Fig. 1C). Nevertheless, endogenous MBD2 is required for expression of *hsa-mir-496* cells in both MCF-7 and MDA-MB-231 cells since depletion of MBD2 results in concomitant reduction of *hsa-mir-496* expression *(* *Fig. 1D* *)*.

**Table 2:** microRNAs that become demethylated upon overexpression of MBD2 in MCF-10A as determined by meDIP array.

microRNA	Differential Methylation	p-value
hsa-mir-369/hsa-mir-409/hsa-mir-410/hsa-mir-412	−1.84488999	0.003090962
hsa-mir-LET7D	−1.705679801	0.002119279
hsa-mir-369/hsa-mir-409/hsa-mir-410/hsa-mir-412	−1.467069246	0.00059518
**hsa-mir-496**	**−1.108922459**	**0.002357951**
hsa-mir-7-1	−1.029150729	0.005266684
hsa-mir-554	−1.008874183	0.000642931
hsa-mir-487A	−0.939064743	0.005950832
hsa-mir-195/hsa-mir–497	−0.873277823	0.004407324
hsa-mir-556	−0.85831035	0.003958848
hsa-mir-138-1	−0.826516597	0.003580814
hsa-mir-299/hsa-mir-411	−0.798663597	0.005423464
hsa-mir-1258	−0.664067098	0.005363877
hsa-mir-30B	0.829194578	0.005779723
hsa-mir-1197/hsa-mir-323/hsa-mir-329-1/hsa-mir-380/hsa-mir-758	0.884900076	0.005776804
hsa-mir-105-1	0.902827667	0.002761197
hsa-mir-145	0.929830403	0.004043383
hsa-mir-181A1/hsa-mir-181B1	0.952147603	0.002793798
hsa-mir-376C/hsa-mir-654	0.962272074	0.000988923
hsa-mir-LET7G	0.976118085	0.004514429
hsa-mir-376C/hsa-mir-654	0.993443213	0.001503151
hsa-mir-95	1.018465615	0.005226545
hsa-mir-218-2	1.195031708	0.001063377
hsa-mir-122	1.245473815	0.002277151
hsa-mir-197	1.26353292	0.003891208
hsa-mir-299/hsa-mir-379/hsa-mir-411	1.264605864	0.001428673
hsa-mir-218-2	1.407366769	0.001059035
hsa-mir-376C/hsa-mir-654	1.415468586	0.005711929
hsa-mir-376C/hsa-mir-654	1.437384831	0.004311179
hsa-mir-145	1.927577721	0.001394248
hsa-mir-609	3.152198973	0.002154375

**Figure 2:**
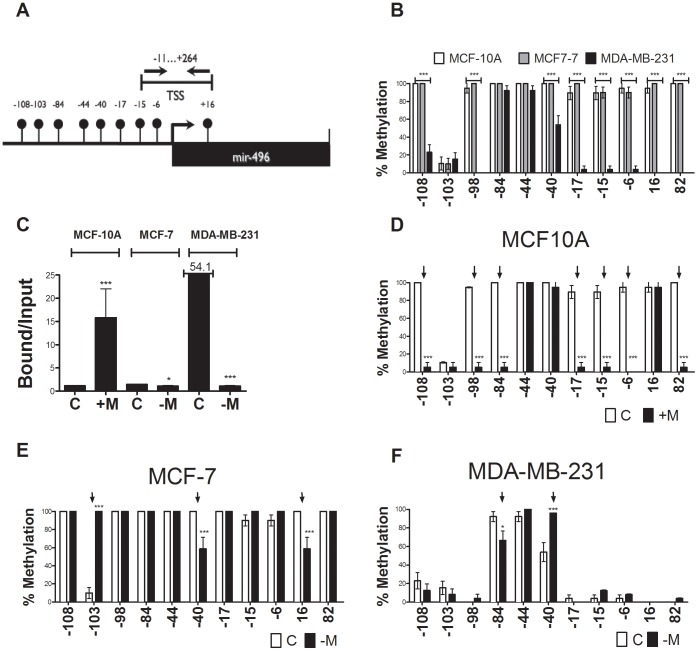
MBD2 overexpression in MCF-10A cells induces *hsa-mir-496* expression and demethylation through binding to the TSS. (A) Physical map of the 5′ region of *hsa-mir-496*. The balloons represent CG dinucleotide sequences. The transcription start site TSS is indicated. The position of primers used to amplify in qChIP analysis the are indicated. (B) Results of bisulfite mapping of the 5′ *hsa-mir-496* in MCF-10A (empty), MCF-7 (grey) and MDA-MB-231 cells (dark) (C) qPCR ChIP of MBD2 in MCF-10A, MCF-7 and MDA-MB-231 with primers as outlined in panel A. MCF-10A [C], MCF-10A expressing ectopic MBD2 [+M], MCF-7 Control [C] and MBD2 depleted MCF-7 cells [-M], Control [C] and MBD2 depleted [-M] MDA-MB-231 cells with primers as outlined in panel A (D) Results of bisulfite mapping of the 5′ *hsa-mir-496* in MCF-10A (empty) [C] transfected with MBD2 (black). (E) Results of bisulfite mapping of the 5′ *hsa-mir-496* in MCF-7 cells (empty) or MBD2 depleted MCF-7 cells (Dark). (F) Results of bisulfite mapping of the 5′ *hsa-mir-496* in MDA-MB-231 cells (empty) or MBD2 depleted MDA-MB-231 cells (Dark).

We then validated the demethylation of *hsa-mir-496* in response to expression of MBD2 as predicted by the DNA methylation array (Fig. 1B). The microRNA *hsa-mir-496* 5′ region upstream to the transcription start site (TSS) (Fig. 2A) is highly methylated in control MCF-10A and MCF-7 cells and is hypomethylated in MDA-MB-231 which express higher levels of *hsa-mir-496* (Fig. 2B) as determined by DNA methylation mapping analysis using bisulfite converted DNA. We show that expression of ectopic MBD2 in MCF-10A cells results in almost complete demethylation of the *hsa-mir-496* promoter (results of bisulfite analysis in Fig. 2D).

To determine whether MBD2 directly interacts with *hsa-mir-496* promoter in the human breast cancer cell line examined, we performed a chromatin immunoprecipitation with an MBD2 antibody as previously described and amplification with primers covering the transcriptional start site (TSS) [15]. Our assay demonstrates binding of MBD2 within the (TSS) (−11–+246) of hsa-mir-496 that correlates with MBD2 expression in the different cell lines (Fig. 2C). MBD2 binding to the hypomethylated hsa-mir-496 promoter in MDA-MB-231 (Fig. 2C) is higher than its binding to the methylated promoter in MCF-10A and MCF-7 cells, consistent with a role for MBD2 in interacting with an active and demethylated *hsa-mir-496* (Fig. 2C). Ectopic expression of MBD2 results in dramatically increased binding of MBD2 to the region proximal to the TSS (Fig. 2C) and loss of DNA methylation of the same region (specifically CpGs at −17,−15, and −6 are demethylated in MCF-10A cells transfected with MBD2) (Fig. 2D).

To determine whether endogenous MBD2 plays a role in *hsa-mir-496* DNA methylation we measured the effects of depletion of *MBD2* mRNA in two breast cancer cell lines MCF-7 and MDA-MB-231 that express significant levels of MBD2 (Fig. 1C). *Hsa-mir-496* is highly methylated in MCF-10A cells and we reasoned that depletion of MBD2 in these cells would have very little further impact on DNA methylation. SiRNA knock down achieved significant MBD2 depletion of 80% in MCF-7 and 60% in MDA-MB-231) (Fig. 1C for QPCR) and depletion of MBD2 binding to the TSS of *hsa-mir-496* in both cell lines (Fig. 2C). Following MBD2 knockdown in MDA-231 cells methylation at the *hsa-mir-496* promoter is significantly increased at −40 while remaining sites are relatively hypomethylated following depletion of MBD2 (Fig. 2F). In MCF-7 cells, which express lower levels of MBD2 than MDA-MB-231 cells the *hsa-mir-496* promoter is heavily methylated except at site −103 which is completely hypomethylated. This site is completely methylated in response to MBD2 depletion (Fig. 2E) while sites at −40 and +16, which are fully methylated in these cells, are partially demethylated following MBD2 depletion (Fig. 2E). In summary, endogenous MBD2 depletion results in alteration of the DNA methylation state of *hsa-mir-496* with increased methylation of specific sites as well as demethylation of other sites. Interestingly, different sites are impacted in response to MBD2 depletion in different breast cancer cell lines. However, in both cases MBD2 depletion resulted in concomitant partial reduction in *hsa-mir-496* expression (Fig. 1D; 1.76-fold and 3-fold decrease in MCF-7 cells and MDA-MB-231 respectively). It is unclear whether these partial changes in DNA methylation are involved in the inhibition of MBD2 expression or whether MBD2 has an impact on *hsa-mir-496* expression that is independent of DNA methylation.

### MBD2 activates methylated *hsa-mir-496* promoter activity in a luciferase reporter transient transfection assay

**Figure 3:**
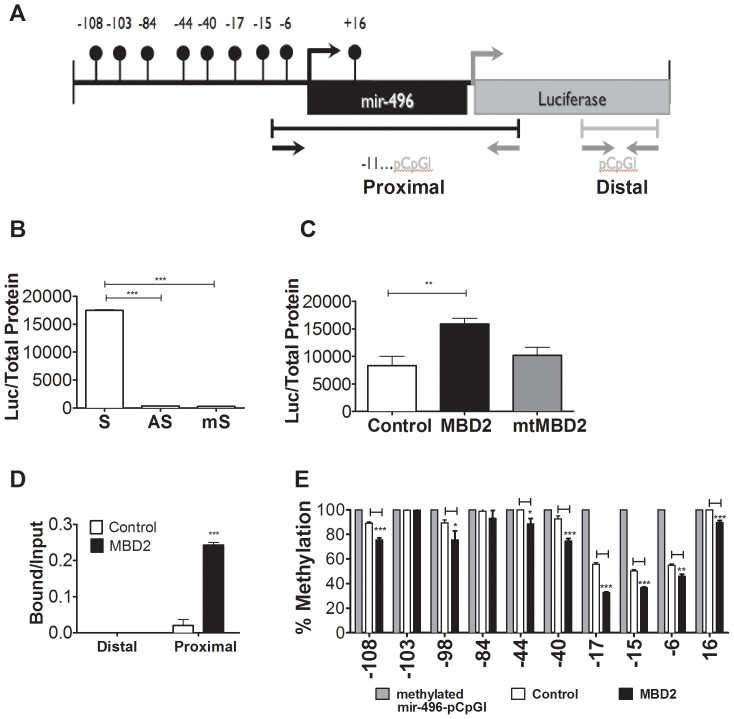
DNA methylation silences *hsa-mir-496* and ectopic MBD2 induces methylated *hsa-mir-496* by transient transfection luciferase assay. (A) Physical map of the *hsa-mir-496*- pCpGl Luciferase reporter. The position of CG dinucleotide sequences are indicated as balloons which are all located in the *hsa-mir-496* 5′ region. Arrows indicate position of primers used for Q-Chip. The position of primer used for pyrosequencing is indicated by a horizontal arrows under the scheme. (B) Relative luciferase activity in HEK 293 cells transiently transfected with *hsa-mir-496* promoter cloned into pCpGl in Sense [S], Antisense [AS] and *in vitro* methylated sense *hsa-mir-496- pCpGl* [mS]. (C) Relative luciferase activity in HEK293 cells co-transfected with methylated *hsa-mir-496- pCpGl* and empty pEF6 vector [Control], MBD2 expression vector [MBD2] or MBD2 mutant without the MBD domain [mtMBD2]. (D) Ectopic MBD2 binding to methylated *hsa-mir-496* region in the *hsa-mir-496- pCpGl* plasmid in transiently transfected HEK 293 cells as determined by QPCR of a ChIP assay with antiMBD2 antibody. The position of primers used for amplification is indicated in (A). (E) Bisulfite pyrosequencing of methylated *hsa-mir-496-pCpGl* (grey), MBD2 immunoprecipitation and bisulfite sequencing of *hsa-mir-496* -pCpGl following transient-co-transfection experiment of methylated *hsa-mir-496* with either pEF6 plasmids (empty) or pEF-MBD2 expression vector (black) in HEK-293 cells.

Our data derived from forced expression of MBD2 in MCF10A cells suggested that MBD2 expression could activate and lead to demethylation of *hsa-mir-496* (Fig. 1 and 2). To directly test the hypothesis that MBD2 could bind and activate the *hsa-mir-496* promoter and alter its state of methylation we used a transient transfection reporter assay. The *hsa-mir-496* region overlapping the predicted TSS, whose state of methylation was examined by bisulfite mapping in Fig. 2B–F, was cloned into CpG-free pCpGl –luciferase reporter (hsa-mir-496-pCpGl) (Fig. 3A). This construct has CG DNA methylation target sequences only in the *hsa-mir-496* promoter region and the assay is therefore not confounded by vector DNA methylation and measures directly the effects of DNA methylation and MBD2 on the transcriptional activity of this region as well its state of methylation. We first demonstrate that this region directs transcriptional activity as indicated by the fact that the region cloned in the sense orientation directed 45-fold higher luciferase activity than in the antisense direction (Fig. 3B). *In vitro* methylation of all CGs in this region with Sss1 DNA methyltransferase which recapitulates the situation in MCF-10A cells reduced luciferase activity 59-fold relative to the unmethylated control supporting the conclusion that methylation of CG sites in the *hsa-mir-496* promoter region silenced its activity (Fig. 3B). The transcriptional activity of methylated *hsa-mir-496*-pCpGl increases when the methylated reporter is co-transfected with MBD2 expression vector as compared with an empty backbone (pEF6) (1.8 fold) and does not increase with an MBD2 methylated DNA binding domain (MBD) deletion mutant (mtMBD2) (Fig. 3C). A ChIP assay with an anti-MBD2 antibody demonstrated that MBD2 directly interacts with the ectopic *hsa-mir-496*-promoter region (our primer set selectively amplifies ectopic *hsa-mir-496* (see description in materials and methods) but not to a distal region in the vector, which served as a negative control (Fig. 3D).

We then tested whether the binding of MBD2 to ectopic *hsa-mir-496* promoter results in a change in its state of methylation. Any demethylation detected must be active since the plasmid is transiently transfected and does not contain an origin of replication. We captured the ectopic *hsa-mir-496* promoter DNA molecules that were interacting with MBD2 (in both empty vector transfection where the transfected *hsa-mir-496* was interacting with endogenous MBD2 and ectopic MBD2 transfectants which had an excess of ectopically transfected MBD2) by ChIP using anti MBD2 antibody and treated the captured DNA with sodium bisulfite. The bisulfite converted ectopic *hsa-mir-496* promoter was amplified with specific primers (that don't amplify the endogenous gene, see Table 1) and was subjected to pyrosequencing. The results presented in Fig. 3E show that sites − 44,−40,−17,−15,−6 and +16 in methylated-*hsa-mir-496-pCpGl* were demethylated in endogenous MBD2 bound DNA (Fig. 3E, empty and dark box) when compared to naked methylated DNA (grey box). Ectopic expression of MBD2 increased demethylation at −108, −98, −40, −17,−15, and −6 and +16 suggesting that increasing levels of MBD2 over endogenous levels enhanced the extent of demethylation of transiently transfected *hsa-mir-496 promoter* bound to MBD2 (Fig. 3E dark box). Since our assay measures demethylation in molecules that are physically bound to the MBD2 protein, these results show that binding of MBD2 to the *hsa-mir-496* promoter is associated with site specific demethylation.

### Genes silenced in response to MBD2 overexpression are targets of *hsa-mir-496*

**Figure 4:**
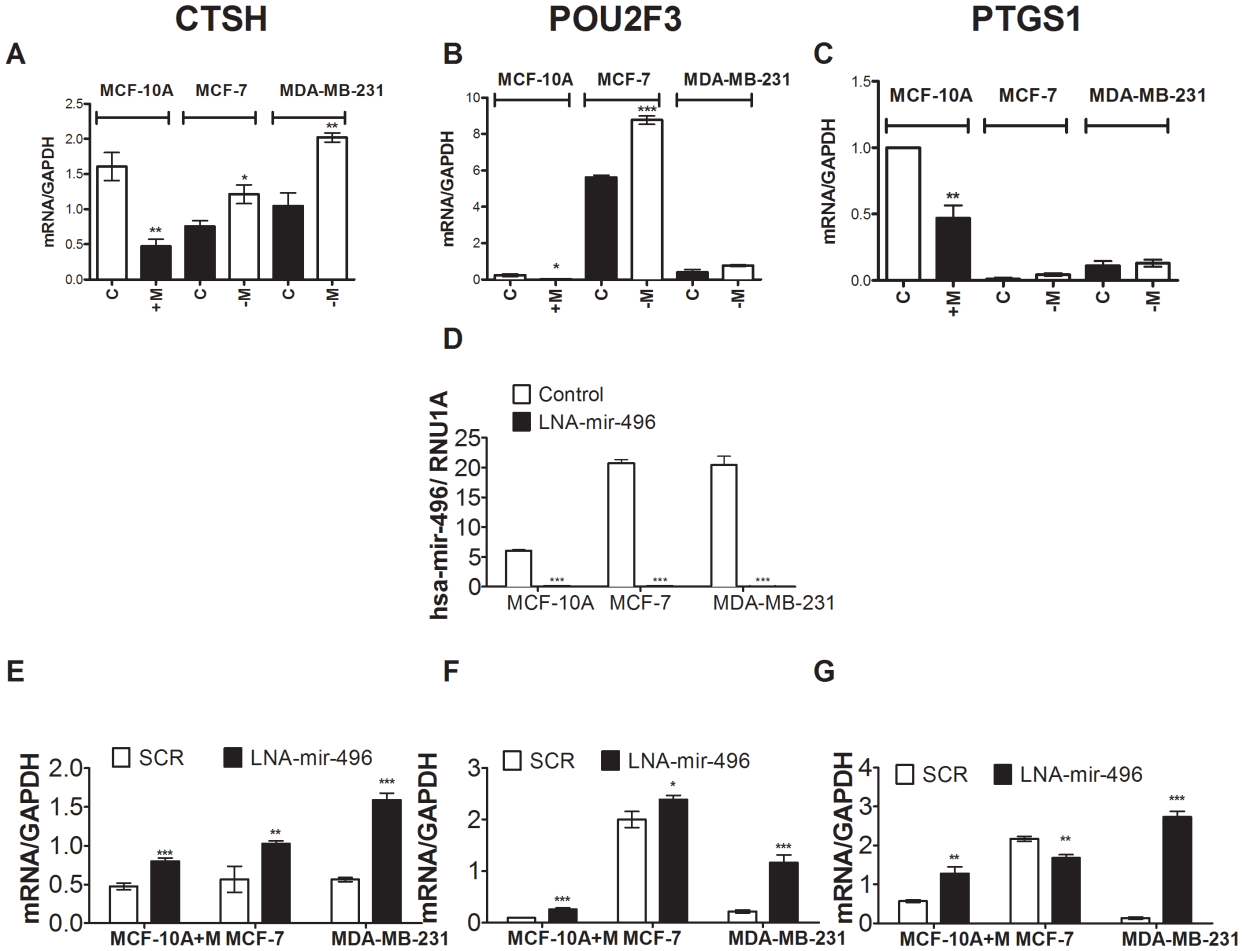
Repressed targets of MBD2 in MBD2 overexpressing cells are putative targets of *hsa-mir-496*. (A) *CTSH* expression in MBD2 overexpressing MCF-10A [+M] and siMBD2 depleted MCF-7 [−M ] and MDA-231 cell lines and controls [C]. (B) *POU2F3* expression in MBD2 overexpressing MCF-10A [+M] and in response to transient depletion of MBD2 [−M] in MCF-7 and MDA-231 and controls [C]. (C) *PTGS1* expression in MBD2 overexpressing MCF-10A [+M] and in response to transient depletion of MBD2 [−M] in MCF-7 and MDA-231 and controls [C]. (D) *hsa-mir-496* expression as determined by QPCR analysis in LNA treated MCF-10A, MCF-7 and MDA-MB-231 cells. (E) *CTSH* expression in response to transient knockdown of *hsa-mir-496* in MCF-10A overexpressing MBD2 , MCF-7 and MDA-231 and controls . (F) *POU2F3* expression in a transient knockdown of *hsa-mir-496* in MCF-10A overexpressing MBD2 , MCF-7 and MDA-231 and controls. (G) *PTGS1* expression in a transient knockdown of *hsa-mir-496* in MCF-10A overexpressing MBD2 , MCF-7 and MDA-231 and controls.

We tested the hypothesis that MBD2 would repress specific genes through activation of microRNA. *in silico* scanning using mirANDA identified a set of predicted targets of *hsa-mir-496*. We then tested whether some of these genes were silenced in MCF-10A cells that overexpressed MBD2 (Table S2)by examining an Affymetrix gene expression array of control and MBD2 overexpressing MCF-10A cells.In a preliminary scan we examined by qPCR 20 mRNAs. We then tested whether these 20 target mRNAs would also be affected by changes in MBD2 in other cell lines. Three of these 20 genes that are *in silico* targets of *hsa-mir-496* that were found to be silenced in response to MBD2 overexpression were also regulated by MBD2 in other cell lines: *Cathespin H (CTSH), POU domain class 2 transcription factor 3(POU2F3)* and *prostaglandin-endoperoxide synthase 1(PTGS1)*. *CTSH*, *POU2F3* and *PTGS1* were all down regulated in MCF-10A cells in response to MBD2 overexpression (3.4-fold, 10.7-fold and 2.13-fold respectively Fig. 4 A–C). Upon endogenous MBD2 depletion *CTSH* and *POU2F3* were upregulated in MCF-7 (1.61-fold and 1.56-fold, respectively) and MDA-MB-231 cells (1.93-fold, and 1.91-fold respectively) (Fig. 4A–C). This supports the conclusion that endogenous MBD2 is indeed involved in silencing of these genes.

We then determined whether the effect of up or down regulation of MBD2 on expression of these genes was mediated by *hsa-mir-496*. We depleted *hsa-mir-496* with a locked nucleotide antisense oligonucleotide targeting *hsa-mir-496* (using a scrambled LNA as a control) in MCF-10A that overexpress ectopic MBD2 as well as MCF-7 and MDA-MB-231 cells which express endogenous MBD2 (Fig. 4D). Following *hsa-mir-496* knockdown, *CTSH, POU2F3* were induced in all three cell lines as expected if these genes are downregulated by *hsa-mir-496* (Fig. 4C,E) while *PTGS1* was induced in MCF-10A cells overexpressing MBD2 and MDA-MB-231 cells expressing high level of MBD2 but not in MCF-7 cells (Fig. 4G). These data are consistent with the hypothesis that *CTSH* and *POU2F3* suppression by MBD2 is mediated by *hsa-mir-496* while *PTGS1* regulation by MBD2 seems to involve other factors in MCF-7 cells.

### Down regulated mRNAs with MBD2 overexpression identify networks of putative targets of *hsa-mir-496*

**Figure 5:**
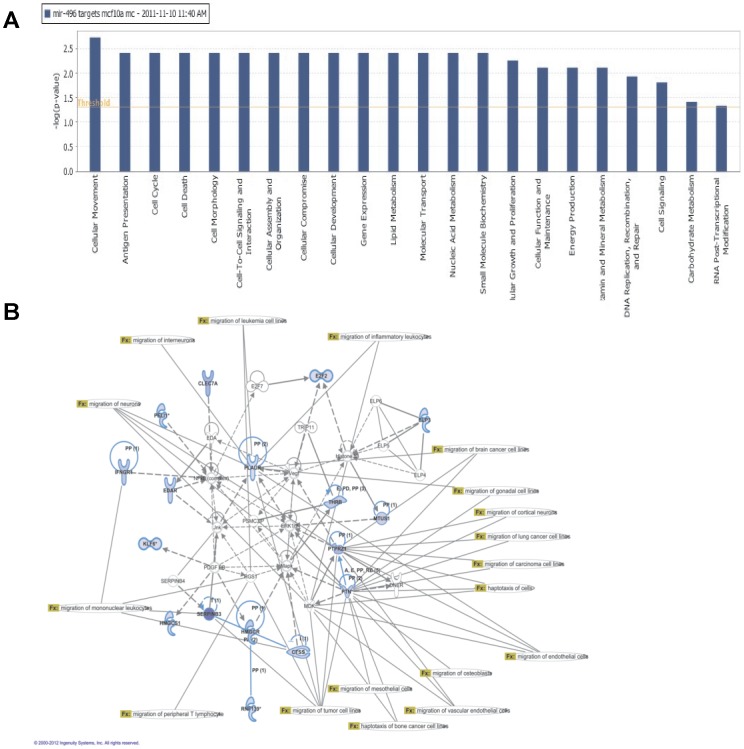
Ingenuity pathway analysis of putative targets of the MBD2*-hsa-mir-496* pathway in MCF-10A cells overexpressing MBD2. (**A**) A list of genes repressed by MBD2 overexpression in MCF-10A cells was compared to a computed list of *hsa-mir-496* targets (miRANDA) and subjected to Ingenuity pathway analysis. (B) Associated network functions identified a network with a role in cell migration and haptotaxis. Down regulated mRNA and putative *hsa-mir-496* targets are highlighted in bold and light blue outline. Data were analyzed through the use of IPA (Ingenuity® Systems, www.ingenuity.com).

Expression analysis of mRNA in MCF-10A stably overexpressing MBD2 identified 5129 genes that were significantly (p<0.005) repressed (<0.9 ratio fold change) in comparison with control MCF-10A cells (Table S1). Cross-referencing this list with a computed list of putative *hsa-mir-496* targets (using miRANDA) identified a dataset of 141 (Table S2) genes repressed by MBD2 that are putative targets of *hsa-mir-496*. We used Ingenuity Pathways Analysis (IPA) suite to delineate the gene networks that the genes in the list fall into. IPA identified networks with a unique role in cell movement, antigen presentation, cell cycle and cell death (Fig. 5A). Associated network functions identified down regulated mRNAs integral to pathways that promote migration (p =  6.79E-5 - 9.1E-5) and haptotaxis (p = 8.62E-5 - 2.94E-2) (Fig. 5B). Interestingly, down regulation of MBD2 was previously shown to reverse invasiveness and metastasis in breast cancer [37] and prostate cancer cell lines [14].

## Discussion

The *cis* silencing of gene expression by DNA methylation is very well documented. Methylated DNA binding proteins including MBD2 bind methylated CGs and recruit chromatin repressor complexes that silence in *cis* gene expression [7]. The data presented here delineates a potential novel pathway of gene regulation by MBD2 that amplifies a DNA methylation signal by affecting downstream genes through mechanisms that don't necessarily require *cis*-DNA methylation in the affected genes.

We provide here several lines of evidence that in combination provide strong support for this hypothesis. We show first that ectopic MBD2 expression in untransformed epithelial cells results in upregulation of a microRNA *hsa-mir-496* (Fig. 1D). Second, expression of *hsa-mir-496* is partially dependent on endogenous MBD2 in two breast cancer cell lines; partial depletion of MBD2 results in reduction in *hsa-mir-496* expression (Fig. 1D). Third, overexpression of MBD2 in MCF-10A cells triggers demethylation of the promoter of *hsa-mir-496* (Fig. 2D). Fourth, MBD2 interacts with chromatin at the *hsa-mir-496* promoter as determined by a ChIP assay, which is consistent with the hypothesis that MBD2 activates *hsa-mir-496* directly *in cis* rather than the alternative hypothesis that MBD2 activation of *hsa-mir-496 is* mediated through MBD2 repressive activity on a putative *trans* acting repressor gene (Fig. 2B). Fifth, a luciferase reporter assay demonstrates that the *hsa-mir-496* promoter region *per se* is silenced by DNA methylation and that it is activated with ectopic expression of MBD2 (Fig. 3 B and C). Sixth, MBD2 targets and binds an ectopic *hsa-mir-496* promoter (Fig. 3D). This provides further evidence for direct action of MBD2 on the *hsa-mir-496* promoter as an MBD2 antibody pulls down the *hsa-mir-496* DNA region and not other regions on the vector. Seventh, the physically MBD2 bound ectopic *hsa-mir-496* promoter molecules are partially demethylated delineating a tight relationship between MBD2 binding and DNA demethylation of the *hsa-mir-496* promoter (Fig. 3E). Taken together these results are consistent with the hypothesis that MBD2 could regulate *hsa-mir-496* promoter activity and its DNA methylation state.

Although our data shows that interaction of ectopic MBD2 with the *hsa-mir-496* promoter results in demethylation, our data does not directly demonstrate that MBD2 is demethylating *hsa-mir-496* promoters in cells nor does it claim that MBD2 is involved in its demethylation. Our data is also consistent with the alternative possibility that MBD2 interaction with this promoter results indirectly in recruitment of other mechanisms that were recently proposed to cause DNA demethylation such as complexes including TET enzymes and base excision repair activity (BER) [38] or hydroxymethylation mediated [39] or direct demethylation by DNMTs [40]. Although we have no evidence for these indirect mechanism in the case of *hsa-mir-496* promoter, further experiments are required to test this hypothesis that are beyond the scope of this paper.

We also show in two breast cancer cell lines that endogenous MBD2 is required for expression of *hsa-mir-496* since depletion of MBD2 results in reduction of *hsa-mir-496* expression (Fig. 1D). However, not surprisingly the expression of *hsa-mir-496 in these cells* is not determined exclusively by the levels of MBD2. MCF10A cells express higher levels of *hsa-mir-496* than MCF-7, which have higher steady-state levels of MBD2 (Fig. 1 A, C, D). Genes are known to be regulated by networks of factors that differ from cell type to cell type, nevertheless in both cell lines expression of MBD2 is required for expression.

In contrast to over expression of MBD2 in MCF-10A cells which results in a dramatic hypomethylation, depletion of MBD2 in MCF-7 and MDA-MB-231 cells results in very limited hypermethylation in spite of a significant decrease in expression of *hsa-mir-496.* This is consistent with the conclusion that MBD2 is required for *hsa-mir-496* activity independently of the state of methylation. However, it is also possible that our assay wasn't sensitive enough to detect partial changes in DNA methylation in response to partial depletion of MBD2. The fact that MBD2 depletion results in both increase and decrease in DNA methylation of different sites suggests that MBD2 has a complex effect on the state of methylation.

A single microRNA has several hundred putative targets in the transcriptome. This allows for amplification and coordination of gene regulation events in the cell. Thus, activation of one microRNA could result in suppression of several RNAs. It has been previously shown that DNA methylation regulates expression of several microRNAs and that hypermethylation of microRNA silences them particularly in cancer leading to gene activation [41]–[43]. However, a microRNA dependent pathway of suppression of gene expression by activation of a microRNA by a methylated DNA binding protein has not been described before. The presence of such a pathway can explain the paradoxical observation that treating cells with DNA methylation inhibitors could result in not only in gene induction but gene silencing as well.

Although some reports have indicated a role of *hsa*-*mir-496* in alcohol exposure [44], and aging [45] its expression has not yet been functionally linked to any repressed targets. By comparing the list of repressed genes in response to MBD2 in MCF-10A cells from an expression array and *in silico* predicted *hsa-mir-*496 targets we derived possible gene targets for MBD2*-hsa-mir-496* pathway. We focused on the genes *CTSH, POU2F3* and *PTGS1* since they were validated to be repressed by increased MBD2 levels in MBD2 transfected MCF-10A cells. Furthermore CTSH and POU2F3 were then shown to be induced by depletion of MBD2 in MDA-MB-231 cells and MCF-7 cells (Fig. 4A,B,C). *Hsa-mir-496* is required for MBD2 repression of these genes since depletion of *hsa-mir-496* (Fig. 4D) in MBD2 overexpressing MCF-10A cells results in relief of repression (Fig. 4E, F,G). *Hsa-mir-*496 depletion in MCF-7 and MDA-MB-231 results in induction of *CTSH* and *POU2F3* (Fig. 4E,F). *PTGS1* is induced in MDA-MB-231 cells by *hsa-mir-496* depletion (Fig. 4G) but not in MCF-7 cells suggesting that other factors regulate this gene in MCF-7 cells (Fig. 4G). Conversely, although *PTGS1* is responsive to *hsa-mir-496* depletion in MDA-MB-231 cells (Fig. 4G) it is not affected by MBD2 depletion in these cells (Fig. 4C). This could be explained by insensitivity of *PTGS1* to the extent of reduction of *hsa-mir-496* that is brought about by partial *MBD2* depletion in MDA-MB-231 cells using siMBD2 treatment (Fig. 1D).

MBD2 was previously implicated in cancer growth and metastasis in several types of cancers [13]–[17] including breast cancer [13]. Part of this effect is mediated through activation of prometastatic genes that is associated with their demethylation. Although targets of MBD2 have been identified to play functional roles in cancer, few have identified targets that exert direct higher-order regulation such as a microRNAs or transcription factors. microRNAs have been extensively characterized to be hypermethylated in different cancers [41]–[43]. The results described here offer a different mechanism by which MBD2 could affect gene expression in cancer and other physiological states: coordinated repression of genes through activation of microRNA.

While here we present a case for the activation of a microRNA through binding of a methylated DNA binding protein that involves promoter demethylation, it most probably involves several other mechanisms including recruitment of transcriptional machineries [46], [47]and histone complexes [48]. Further experiments are required to unravel these additional events regulating *hsa-mir-496* expression in different cell types.

The genes that were validated here as targets of *hsa-mir-496* are known to be involved in different aspects of cancer progression. *CTSH* (Cathespin H) is a cysteine protease whose activity is often upregulated during cancer metastasis [49]. It is surprising therefore that this gene is down regulated by MBD2 and *hsa-mir-496* in highly invasive breast cancer cells MDA-MB-231. It is clear however that what defines the metastatic state is not a select list of genes but a complex network and the overall output of the network. On the other hand downregulation of *CTSH* has been observed during osteolysis in highly metastatic breast cancers which is consistent with a role in promoting metastasis [50]. *POU2F3 i*s a transcription factor that has been largely silenced in cervical cancer [51] and has been highlighted as a tumor suppressor gating the transformation of primary cell lines to metastatic melanomas [52]. Its silencing by overexpression of MBD2 through *hsa-mir-496* is consistent with a role in cancer. These data suggest a different mechanism for suppression of tumor suppressors in cancer than the known mechanism of suppression by *cis* DNA methylation; long-range suppression through demethylation of regulatory microRNA. *PTGS1* is involved in prostaglandin synthesis and it is deregulated in pancreatic cancer [53].

A limitation of our studies is that we only used mir-496 antagonists in the current study. Follow up studies should focus on other assays to strengthen this relationship (3′UTR assays, exogenous mir-496, etc.).

In addition to experimentally validating several targets of *hsa-mir-496*, by cross-referencing *of hsa-mir-496 in silico* targets with down regulated mRNAs in MBD2 overexpressing MCF-10A cells we derived a list of 141 genes whose repression is potentially downstream to the MBD2-*hsa-mir-496* pathway. Ingenuity Pathway analysis of this list revealed highly significant functional gene networks involved in cellular movement, cell cycle, cell death and antigen presentation (Fig. 5A). These are molecular pathways that are potentially involved at different stages of cancer progression. Within this subset we looked directly at the pathways of down regulated mRNAs and putative targets of *hsa-mir-496* to identify a possible role in migration and haptotaxis. Future studies need to test the hypothesis that this is a mechanism for a coordinated repression of important gene networks in cancer by DNA methylation regulators such as MBD2.

**Figure 6:**
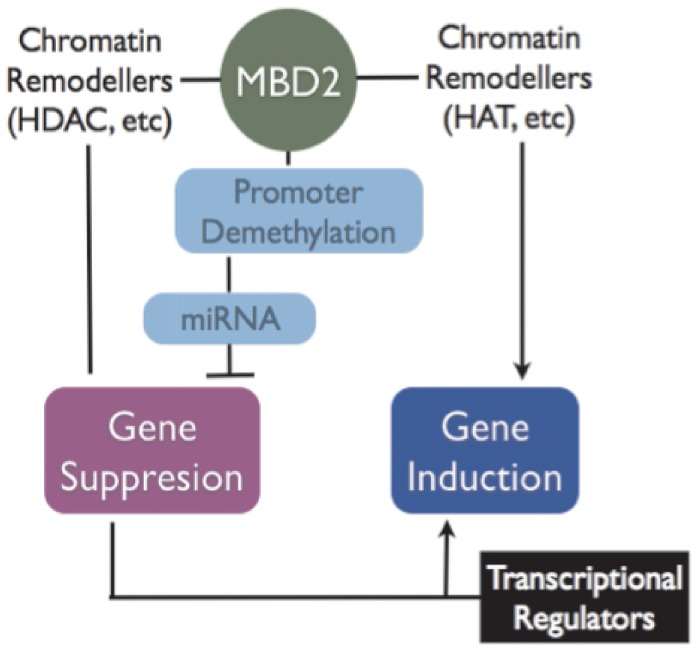
MBD2 mediated repression through the direct activation of microRNA a model. MBD2 represses methylated genes in *cis* by recruiting chromatin repressor complexes. It can also induce gene expression through demethylation or recruitment of chromatin activation complexes. A new pathway for long-range repression mediated through activation and demethylation of microRNA is supported by the data presented in this paper.

In summary, our data points to the intricate ways by which DNA methylation and its binding proteins could regulate gene expression. Several genome-wide studies have tried to correlate overall gene expression patterns and *cis*-DNA methylation states. Invariably, these are not perfect correlations. Although these inconsistencies could easily be explained by DNA methylation independent mechanisms, our data shows that *bona fide* DNA methylation regulators such as MBD2 could trigger a sequence of gene expression events downstream from the initial *cis* acting DNA methylation signals (Fig. 6 for model). The data illustrates how a DNA methylation signal in a single region could be amplified and affect multiple downstream targets without necessarily altering their state of methylation. If the targets fall into discrete functional pathways (Fig. 5) this mechanism could coordinate responses to single DNA methylation regulators such as MBD2. Similarly, pharmacological demethylation as well as global hypomethylation in cancer and other diseases must not result in gene induction exclusively as is commonly thought, but could result in gene repression as well through pathways such as the one delineated here. Future computations of the impact of DNA methylation in genome-wide and transcriptome wide studies need to take into account these hierarchies of gene expression-repression that could be triggered by single demethylation events.

## Supporting Information

Table S1 **Repressed transcripts following MBD2 overexpression in MCF-10A.**
(XLSX)

Table S2 **Repressed transcripts following MBD2 overexpression in MCF-10A that overlap with putative targets of hsa-mir-496.**
(XLSX)
